# The effectiveness of interventions aimed at increasing physical activity in adults with persistent musculoskeletal pain: a systematic review and meta-analysis

**DOI:** 10.1186/s12891-017-1836-2

**Published:** 2017-11-22

**Authors:** Joanne Marley, Mark A. Tully, Alison Porter-Armstrong, Brendan Bunting, John O’Hanlon, Lou Atkins, Sarah Howes, Suzanne M. McDonough

**Affiliations:** 1 Centre for Health and Rehabilitation Technologies, Institute of Nursing and Health, School of Health Sciences, Ulster University, Shore Road, Newtownabbey, Co Antrim BT37 0QB UK; 2 Centre for Public Health, Queens University Belfast, Royal Victoria Hospital, Grosvenor Road, Belfast, BT12 6BA UK; 3 UKCRC Centre of Excellence for Public Health (Northern Ireland), Centre for Public Health, School of Medicine, Dentistry and Biomedical Sciences, Queens University Belfast Room 02020, Institute of Clinical Science B, Royal Victoria Hospital, Grosvenor Road, Belfast, BT 12 6BJ UK; 4 Belfast Health and Social Care Trust, Chronic Pain Service, Belfast City Hospital, 51 Lisburn Road, Belfast, BT9 7AB UK; 5 Centre for Behaviour Change, University College London, 1-9 Torrington Place, London, UK; 6 Honorary Research Professor, School of Physiotherapy, University of Otago, Dunedin, New Zealand

**Keywords:** Physical activity, Low back pain, Osteoarthritis, Musculoskeletal pain, Chronic pain, Persistent pain, Behaviour change techniques, Systematic review

## Abstract

**Background:**

Individuals with persistent musculoskeletal pain (PMP) have an increased risk of developing co-morbid health conditions and for early-mortality compared to those without pain. Despite irrefutable evidence supporting the role of physical activity in reducing these risks; there has been limited synthesis of the evidence, potentially impacting the optimisation of these forms of interventions. This review examines the effectiveness of interventions in improving levels of physical activity and the components of these interventions.

**Methods:**

Randomised and quasi-randomised controlled trials were included in this review. The following databases were searched from inception to March 2016: CENTRAL in the Cochrane Library, Cochrane Database of Systematic Reviews (CDSR), MEDLINE, Embase, CINAHL, PsycINFO and AMED. Two reviewers independently screened citations, assessed eligibility, extracted data, assessed risk of bias and coded intervention content using the behaviour change taxonomy (BCTTv1) of 93 hierarchically clustered techniques. GRADE was used to rate the quality of the evidence.

**Results:**

The full text of 276 articles were assessed for eligibility, twenty studies involving 3441 participants were included in the review. Across the studies the mean number of BCTs coded was eight (range 0–16); with ‘goal setting’ and ‘instruction on how to perform the behaviour’ most frequently coded. For measures of subjective physical activity: interventions were ineffective in the short term, based on very low quality evidence; had a small effect in the medium term based on low quality evidence (SMD 0.25, 95% CI 0.01 to 0.48) and had a small effect in the longer term (SMD 0.21 95% CI 0.08 to 0.33) based on moderate quality evidence. For measures of objective physical activity: interventions were ineffective - based on very low to low quality evidence.

**Conclusions:**

There is some evidence supporting the effectiveness of interventions in improving subjectively measured physical activity however, the evidence is mostly based on low quality studies and the effects are small. Given the quality of the evidence, further research is likely/very likely to have an important impact on our confidence in effect estimates and is likely to change the estimates. Future studies should provide details on intervention components and incorporate objective measures of physical activity.

**Electronic supplementary material:**

The online version of this article (10.1186/s12891-017-1836-2) contains supplementary material, which is available to authorized users.

## Background

Epidemiological studies suggest one in five people across Europe suffer from persistent pain [1, 2]. Most persistent pain arises from musculoskeletal disorders, such as low back pain and osteoarthritis; both of which are considered leading causes of disability, worldwide [3]. It can be expected that with aging populations, the health, economic, and social problems associated with these conditions are likely to rise [1, 2, 4]. In addition to causing considerable disability, persistent musculoskeletal pain (PMP) also increases an individual’s risk of developing other health conditions including; depression, obesity, heart disease [5–7], cancer [8] and indeed early mortality [7–9]. Despite this, efforts to address these broader health implications of PMP are somewhat lacking.

### Description of the intervention

Clinical guidelines widely endorse exercise and/or physical activity (PA) in the management of PMP [10–17]. This is largely due to the positive impact these interventions can have on reducing pain and disability. However, improving levels of PA can lead to broader health benefits: with even small changes in PA levels leading to substantial health gains [18, 19].

PA can be defined as any movement produced by skeletal muscles resulting in energy expenditure, it occurs across several domains including: social and domestic activities, commuting, recreational and leisure activities [20]. PA may or may not include exercise: exercise is a subset of PA tending to be planned, structured or repetitive [20] with a specific purpose such as improving strength, it has been recommended that the terms PA and exercise are not confused [21].

### How the intervention might work

Improving levels of PA requires behaviour change. Behaviour change interventions are coordinated sets of activities designed to change specified patterns of behaviour [22]. Behaviour change techniques (BCTs) are the components of interventions that effect change [23]. Taxonomies of BCTs have been used to describe intervention content in a number of PA behaviour change interventions [24–28]. Across these interventions and in line with NICE recommendations for individual level behaviour change [29], some consistent techniques appear to be associated with effective interventions e.g. self-monitoring behaviour, providing feedback, and goal setting.

### Why it is important to do this review

PA and exercise interventions are often recommended in the management of PMP as they can have a positive effect on pain and disability levels. However, the extent to which these interventions actually result in changes to behaviour and consequently increased levels of physical activity is less clear. Although individual studies have demonstrated it is possible to increase PA levels in those with back pain [30] or osteoarthritis [31, 32], the results of systematic reviews are conflicting and limited. In adults with osteoarthritis a systematic review concluded that self-management programmes achieve small improvements in subjectively measured PA in the short-term [32]: whereas, a review of PA interventions in adults with PMP reported no improvements in objectively measured PA [33]. Furthermore, the BCTs used within these forms of interventions and the relationship if any, to outcomes has not yet been systematically explored.

### Objectives

This systematic review investigated the effectiveness of any form of intervention with a clear aim of increasing PA in adults with PMP. Possible associations between BCTs or intervention characteristics and intervention effects were also investigated.

The objectives of this review are to:

1. Determine the effectiveness of interventions in increasing PA levels in adults with PMP.
2. Identify BCTs used within interventions.
3. Determine if particular BCTs or other intervention characteristics (intensity, recruitment route, type of PA, etc.) are associated with greater effect sizes.

## Methods

The full protocol for this review has been published [34].

### Population

Randomised and quasi-randomised controlled trials in adults (≥18) with PMP (pain lasting ≥3 months), in the axial skeleton or large peripheral joints were included. We excluded studies focusing on fibromyalgia, inflammatory and/or autoimmune disorders and perioperative patients, which may require a different management strategy.

### Types of interventions

All interventions that had a clear aim of increasing PA in adults with PMP were eligible for inclusion. We excluded site specific rehabilitative exercise interventions unless it was clear the intervention also addressed habitual PA. We included trials with a comparative control group and trials with multiple intervention arms. We did not include population or community-wide interventions.

### Types of outcome measures

The primary outcome of interest was PA measured by self-reported or objective measures; questionnaires, recall diaries, pedometers or actigraphy. Measurements of adherence or attendance at classes alone, were not sufficient. The secondary outcome of interest was adverse incidents.

### Search methods for identification of studies

Search strategies were developed for each electronic database and were based on the initial Medical Literature Analysis and Retrieval System Online (MEDLINE) strategy (Additional file 1). We searched the Cochrane Central Register of Controlled Trials (CENTRAL) in the Cochrane Library, the Cochrane Database of Systematic Reviews (CDSR) in the Cochrane Library, Ovid MEDLINE(R) Daily Update, Ovid MEDLINE(R), Ovid MEDLINE (R) - includes new records, not yet fully indexed, Ovid Embase, EBSCO Cumulative Index to Nursing and Allied Health Literature (CINAHL), Ovid PsycINFO, AMED (Allied and Complementary Medicine). All databases were searched from inception to March 2016.

Reference lists of systematic reviews and articles retrieved from the search were scanned for additional references.

### Data collection and analysis

#### Selection of studies

Results from the searches were imported into EndNote (X7) bibliographic software (Thomson Reuters, Philadelphia, PA, USA) and duplicates removed. Titles and abstracts obtained from the search were independently screened by two authors (JM 100%, MAT 70% and SMcD 30%). Articles not meeting the inclusion criteria and outside the scope of the review were removed. Full text reports of the remaining publications were retrieved. Two review authors (JM, SMcD) used a standardised form tested prior to use, to select trials eligible for inclusion. Non-English papers were assessed and, where necessary, translated in part or in full.

#### Data extraction and management

Data was extracted independently by two reviewers (JM, SMcD) using a customised form tested prior to use. Relevant data was extracted for methodological issues, intervention characteristics, study design, study characteristics and adverse events. Intervention content was coded according to the BCTTv1 [35]. Two coders (JM, SH) independently coded BCTs, inter-rater reliability was assessed using the prevalence-adjusted bias-adjusted Kappa (PABAK) statistic [36]. PABAK adjusted for the high frequency of agreement on absent BCTs. Values of 0.60–0.79 indicated ‘substantial’ reliability and 0.80 and above ‘outstanding’ reliability [37].

#### Assessment of risk of bias in included studies

Two reviewers (JM, SMcD) independently assessed studies for risk of bias (ROB), using the Cochrane risk of bias tool [38]. An additional domain was added to determine if studies were adequately powered. For cluster randomised controlled trials, five additional domains were assessed, as recommended by Cochrane (16.3.2) [38].

#### Quality of the evidence

The Grading of Recommendations, Assessment, Development and Evaluation (GRADE) approach was used to interpret and evaluate the quality of the evidence [39, 40]. The methods and recommendations described in the Cochrane handbook [38] and by the GRADE working group [33] were used to assess the quality of a body of evidence using five domains: risk of bias, inconsistency, indirectness of evidence, imprecision of effect estimates and potential publication bias. Data for each outcome was entered into GRADEpro to create ‘Summary of Findings’ table and footnotes were used to justify all decisions on the downgrading of the quality of the evidence.

The definitions described by the GRADE working group were used to grade the quality of evidence as follows:

- High – Further research is very unlikely to change our confidence in the estimate of effect.
- Moderate – Further research is likely to have an important impace on our confidence in the estimate of effect and may change the estimate.
- Low – Further research is very likely to have an important impact on our confidence in the estimate of effect and is likely to change the estimate.
- Very low – Any estimate of effect is very uncertain.

#### Measures of treatment effect

Continuous outcomes were analysed using post intervention measures, we reported effect sizes using the standardised mean difference (SMD) as outcomes were reported across different scales. For comparisons of the results we categorised studies into effect sizes according to Cohen’s classification; SMD; 0.2 < 0.3 as small, 0.3–0.8 as moderate, >0.8 as large [41]. *P*-values of <0.05 and confidence intervals that excluded null values were considered statistically significant.

#### Unit of analysis issues

Where studies involved multiple intervention groups we followed recommendations suggested by the Cochrane collaboration (16.5.4) [38] by combining similar intervention groups to perform a single pairwise comparison.

Where studies reported PA domains separately or reported more than one PA outcome, data were extracted for each, however, for the effect size analysis, measures of overall PA were given preference, if these were not available leisure time PA was given preference’.

To facilitate exploration of results not suitable for quantitative synthesis we grouped studies by effect size using an aggregate of subjective and objective measures (objective measures given preference to subjective where available) at the post intervention time point.

#### Dealing with missing data

Attempts were made to contact original investigators to request missing data.

The frequency and duration of the intervention was used to calculate an estimated overall intervention contact time ‘intensity’. The calculation was based on the full intervention being delivered as planned. If the duration of a session was not reported or the data was unobtainable from authors, we allocated 20 min for telephone follow up and 45 min for face to face interventions.

#### Assessment of heterogeneity

Diversity across the studies was qualitatively assessed in terms of the intervention, participant demographics, outcome measures and follow-up. Data was assessed for statistical heterogeneity using RevMan version 5.3 using the *I* ^*2*^ statistic, values of *I* ^*2*^ ranging from 30% to 60% were considered to represent moderate heterogeneity and 50% to 90% substantial heterogeneity [38].

#### Data synthesis

Separate meta-analyses were completed for subjective and objective outcome data at three time points; short term (not longer than 12 weeks’ post-randomisation), medium term (not longer than 6 months’ post randomisation) and long term (greater than 6 months post randomisation). Outcomes were analysed using the SMD, with the inverse variance method to calculate the overall effect and standard error, a random effects model was applied to incorporate heterogeneity.

#### Subgroup analysis and investigation of heterogeneity

We performed the following pre-specified subgroup analysis:

- Clinical subgroups: classified as ‘persistent low back pain’ and ‘osteoarthritis’
- Frequency and duration of intervention (intensity) classified as ‘higher’ or ‘lower’ relative to the median number of contact hours across the studies

The following subgroups were planned but not conducted as the data generated was deemed insufficient.

- BCTs
- Recruitment routes

Descriptive statistics were therefore used to explore possible associations between these factors and other intervention characteristics and intervention effects.

#### Sensitivity analysis

A sensitivity analysis was performed to check if excluding studies with a higher ROB affected results. The threshold for sensitivity analysis was set for studies meeting at least 50% of the criteria of the ROB assessment, excluding blinding of participants and providers.

## Results

### Results of the search

**Fig. 1:**
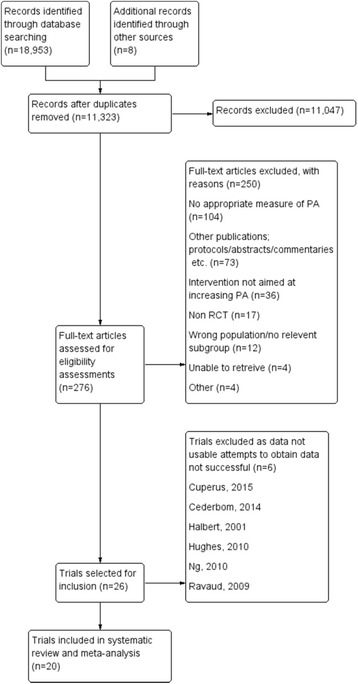
Study Flow Diagram

The electronic searches returned 18,953 records, (Fig. 1) after de-duplication in the referencing software, 11,323 title and abstracts were screened against the inclusion criteria. In total 276 records were identified as potentially relevant, and the full text reports were retrieved. Twenty-six studies were initially agreed for inclusion; six studies were subsequently found to contain unusable outcome data, requests to obtain the data were not successful (Fig. 1). Twenty studies had sufficient data to be included in a meta-analysis [30–32, 42–58]. Nine authors were contacted regarding studies that were deemed to have potentially usable data; six replied, four authors provided the information needed to include their study [43, 44, 52, 58].

Eight non-English language studies were translated but none were eligible for inclusion.

### Excluded studies

A total of (*n* = 250) studies were excluded from the review. Exclusions were most often due to no or unacceptable measures of PA and studies having no clear aim of increasing PA (Fig. 1).

### Characteristics of included studies

Ten studies were described as randomised controlled trials (RCTs), three were cluster RCTs [43, 44, 55], five feasibility or pilot RCTs [30, 32, 48, 51, 56] and one was a controlled clinical trial [42]. Sullivan et al. [57] reported a one year follow-up of patients who had participated in an RCT [59]. The maximum number of groups within studies was three, [44, 46, 47, 52].

### Participants in included studies

The studies involved 3441 suitable participants (4875 in total) (Table 1), over half were female (approx. 59.2%). Thirteen studies focused on osteoarthritis (1874 participants; *n* = 7 knee, *n* = 5 hip and/or knee, *n* = 1 generalised) and seven on persistent low back pain (*n* = 1567 participants). The mean age of participants with osteoarthritis ranged from 61 to 73.8 years, and for persistent low back pain from 40.4 years to 51.9 years.

**Table 1:** Characteristics of included studies (*n* = 20)

Author/Year	Study Design	No of Participants	Gender	Age Range	Condition	Intervention	Control Condition	Recruitment Route	PA Outcome	Longest follow-up
Alaranta, 1994 [42]	Controlled Clinical Trial	293	F160 M133	40.4 (4.8) Control 40.5 (4.6) Intervention	PLBP	Home training programme + Inpatient rehabilitation with education	Inpatient rehabilitation 40–50% less strenuous	Finnish Social Security Insurance Institution	Subjective - Leisure time PA (strenuousness)	12 months
Allen, 2016 [43];	Cluster RCT	300 (patients) 30 (providers)	F28 M272	61.6 (9.2)	OA hip/knee	Patients - Physical activity and weight management counselling Healthcare providers received treatment recommendations	Usual care	Medical records veterans affairs	CHAMPS	12 months (12 month intervention)
Becker, 2008 [44];	Cluster RCT	1378 (chronic pain subgroup 332)	F801 M577 (entire group no figures for subgroup)	49.1 (13.3) guideline group 47.4 (13.5) guideline + MC 50.2 (14.3) Control	LBP (mixed)	Practitioner education – guideline implementation Practitioner education – guideline implementation + MI	Guideline delivered via post	Primary Care GP’s	Freiburg Questionnaire	12 months
Bossen, 2013 [45];	RCT	199	F129 M70	64 (6.6) All 61 (5.9) Intervention 63 (5.4) Control	OA hip/knee	Web based intervention to increase PA using behavioural graded activity	Waiting list	Volunteers from newspapers and websites	PASE and Subgroup ACTi graph	12 months
Brosseau, 2012; [46];	RCT	222	153F 69M	63.9 (103) Walking 63.9 (8.2) Walking + Booklet 62.3 (6.8) Control	OA Knee	Walking group Walking and behavioural education	Self-directed received educational pamphlet	Unclear	7 day Par (recall)	18 months
Farr, 2010;[47];	RCT	293	F218 M75	55.5 (7.3) Resistance training 55.8 (6.1) Self-Management 54.2 (7.3) Combined	OA Knee	Resistance training + self-management	Self-management	General community mass mailings, media ads and local physicians	ACTi graph 7 days	9 months
Focht, 2014;[48];	RCT (pilot)	80	F67 M13	63.5 (6.86)	OA Knee	Group mediated cognitive behavioural exercise intervention	Traditional centre based exercise	Direct referral State Medical Centre Rheumatologists, ads Arthritis Foundation groups	Accelerometer (PA Lifecorder plus) 7 days	12 months
Hiyama, 2012;[49];	RCT	40	32F 19M	71.9 (5.2) Walking 73.8 (5.7) Control	OA Knee	Instructed to increase number of steps, physical therapy + programme of walking	Physical therapy + advice re walking	Unclear - community dwelling females	Pedometer (steps per day)	4 weeks
Hughes, 2006;[50]	RCT (block randomisation)	215	363F 56M	71.1 (59 -91 yrs)	OA hip/knee	Education, exercise and fitness walking	Arthritis self-help book and information on exercise programmes in community	Senior centres, newsletters, local media, presentations to senior groups	Total minutes exercised	12 months
Hunter, 2012;[51];	RCT (feasibility)	51	167F M79	43.2 (13.5) Exercise 42.4 (11.3) Exercise Auricular Acupuncture	PLBP	Exercise and acupuncture	Exercise	Primary Care GP’s, Physiotherapy waiting list and University population	IPAQ (ActivPal - steps per day)	6 months
Hurley, 2015:[52]:	RCT	246	40F	45.4 (11.4)	PLBP	Walking programme Exercise class	Usual physiotherapy	Physiotherapy departments	IPAQ	12 months
Krien, 2013;[53];	RCT	229	29F 200M	51.2 (12.5) Walking 51.9 (12.8) Enhanced Usual Care	PLBP	Walking group	Enhanced usual care	Individuals referred for back class and medical record system	Pedometer (steps per day)	12 months
McDonough, 2013; [30];	RCT (feasibility)	56	31F 25M	51 (42 – 60 yrs) Exercise 48 (43 – 55 yrs) Exercise Walking Programme	PLBP	Education and advice and walking group	Usual care	Physiotherapy waiting lists primary care	MGROC PA (ActivPal - steps per day)	6 months
Meng, 2011;[54];	RCT	360	231F 129M	50.2 (7.6) Intervention 49.5 (7.7) Control	PLBP	Biopsychosocial back school programme (inpatient)	Traditional back school (setting unclear)	Orthopaedic hospital - patients had applied for inpatient rehabilitation	Freiburger Questionnaire	12 months
Pisters, 2010 [55]	RCT Cluster (analysis of secondary outcomes)	200	F154 M46	64.8 (7.9)	OA Hip or Knee	Behavioural graded activity and operant conditioning and exercise therapy	Usual physiotherapy (per clinical guidelines)	Physiotherapists and press releases in local newspapers	PA SQUASH - Converted using METs total hrs. Per week in health enhancing PA	65 weeks (14.9 months)
Schlenk, 2011;[56];	RCT (feasibility)	26	F25 M1	63.2 (9.8)	OA Knee (overweight)	Counselling, exercise, fitness walking programme	Usual care	Rheumatology practices, arthritis disease network registry, self-referral	Diary - Minutes walked per week and other aerobic PA minutes	12 months
Sullivan, 1998;[57];	RCT (follow-up)	102 (52 in this follow-up)	F85 M17 (f44 m8)	70.38 (9.11) Intervention 68.48 (11.32) Control	OA Knee	Supervised fitness walking and supportive education	Standard medical care, weekly interviews about function and daily activity	Community clinics, private clinics - rheumatology	Recall - Average distance walked per week	12 months
Talbot, 2003;[31];	RCT	34	F26 M8	69.59 (6.74) Pedometer 70.76 (4.71) Education	OA Knee	Arthritis self-management programme + walking programme	Arthritis self-management programme	Senior Centres and ads in local papers	Pedometer (steps per day) + Accelerometer	6 months
Trudeau, 2015;[58];	RCT	228 (Subgroup 94)	F72 M156	49.9 (11.6)	Arthritis (all – subgroup data OA spine, large peripheral joints via author)	Web-based painAction programme, informative articles, self-check assessments etc.	Waiting list control	Flyers in surgeries, Pain association members, google adwords, ClinicalTrials.gov. PainEDU.org health professionals	Aerobic exercise minutes (all)	6 months
Williams, 2011 [32]	RCT (feasibility)	119	F76 M43	68.2 (8.1) Intervention 68.6 (8.5) control	OA Hip or Knee	‘New’ Advice booklet – emphasis on addressing exercise related beliefs	Arthritis UK booklet	GP Practices	IPAQ	3 months

*PA* Physical Activity, *MI* Motivational Interviewing, *IPAQ* International physical activity questionnaire, *MGROC* Modified global rating of change (physical activity), *SQUASH* Short questionnaire to assess health enhancing physical activity, *PASE* Physical activity scale for elderly, *7 day PAR* 7-day physical activity recall, *CHAMPS* Community healthy activities model programme for seniors, *OA* Osteoarthritis, *LBP* Low back pain, *PLBP* Persistent low back pain, *RCT* Randomised controlled trial

### Interventions

Table 2 summarises modes of delivery, intervention content, provider and intensity for each intervention. Most studies incorporated more than one mode of delivery but have been described according to what was considered the ‘primary’ delivery mode. Most interventions were provided by healthcare professionals (12/20), other providers included exercise and fitness professionals and a counsellor. Intervention contact times ranged from <1 h for a educational pamphlet [32] to approximately 200 h of contact time [46] occurring over a twelve month intervention. The median number of contact hours was 8.3 h. Walking was the most common form of PA, followed by multicomponent programmes utilising a mixture of aerobic, strengthening and/or general flexibility exercises. All of the interventions incorporated some form of educative component relating to the role of PA in managing PMP.

**Table 2:** Interventions, quality assessment, BCTs - studies grouped post intervention using aggregated outcome measures

Author, Year	Hiyama, 2012;	Hughes, 2006;	Alaranta, 1994;	Focht, 2014;	Pisters, 2010;	Farr, 2010;	Allen, 2016;	Meng, 2011;	Becker, 2008;	Sullivan, 1998;	Williams, 2011;	Brosseau, 2012;	Trudeau, 2015;	Hunter, 2012;	Bossen, 2013;	Schlenk, 2011;	McDonough, 2013;	Krien, 2013;	Hurley, 2015;	Talbot, 2003;
Effect Size SMD 95% CI	1.96 [1.19, 2.73]	0.87 [0.58, 1.15]	0.77 [0.53, 1.01]	0.56 [0.07, 1.06]	0.51 [0.21, 0.80]	0.29 [−0.03, 0.61]	0.28 [0.04, 0.53]	0.25 [0.02, 0.48]	0.17 [−0.07, 0.41]	0.12 [−0.50, 0.74]	0.11 [−0.31, 0.53]	0.10 [−0.27, 0.48]	0.07 [−0.35, 0.49]	0.06 [−0.60, 0.72]	0.02 [−0.50, 0.54]	−0.00 [−0.77, 0.77]	−0.00 [−0.74, 0.73]	−0.03 [−0.35, 0.30]	−0.29 [−0.59, 0.01]	−0.32 [−1.0, 0.35]
ROB assessment	Lower	Higher	Higher	Lower	Lower	Higher	Lower	Lower	Higher	Higher	Lower	Lower	Lower	Lower	Lower	Lower	Lower	Lower	Lower	Higher
*Mode of delivery*																				
Automated Web-based													x		x			x		
Inpatient Programme			x					x												
Centre-based	x	x		x	x	x				x				x		x			x	
Home-based		+	+	x	x												x		x	x
Community-based												x								
Other							x		x		x									
*Session structure*																				
Individual	?	+	+		x	x	x	x	x	+		x				x	x		x (WP)	x
Group based		x	x	x		x		x		x		x		x					x (EC)	x
*Type of PA*																				
Multicomponent Exercise Programme		x	x	x		x		x						x		x			x (EC)	
Walking	x	x		x						x		x				x	x	x	x (WP)	x
User Selected					x		x		x		x				x					
Other/Unclear							x		x		x		x							
*Provider*																				
Physiotherapist	x	x			x					x				x		x	x	+	x	
Nurse									x							x				x
Doctor									x											
Fitness Professional				x		x						x								
Multidisciplinary			x					x												
Other						? SM	?				x		x		x			x		
Estimated Intervention Contact Time (hrs)	3	36	111	36	11.5	134	6	50	1.5	24	0.5	200.5	4.3	8	1.166	7.5	3.5	8.6	8	12.15
No. of BCT’s coded	3	12	3	16	9	5	16	1	0	6	2	14	5	7	12	10	11	8	15	5

*+* to a lesser extent, ? unclear from study description, / not explicit, *SM* self-management, *WP* walking programme, *EC* exercise class., *SMD* standardised mean difference, *CI* confidence intervals, *ROB* risk of bias (meeting at least 50% of domains assessed, excluding blinding participants and providers)

A total of 160 BCTs (mean per study 8, range 0–16) were coded across the 20 studies (Table 2). The most frequently coded techniques were ‘goal setting (behaviour)’ and ‘instruction on how to perform the behaviour’ (65%) followed by ‘behavioural practice/rehearsal’ and ‘self-monitoring of the behaviour’ (55%). A mean PABAK score of (0.9) indicated outstanding agreement on identification of BCTs.

### Control groups

The content of control groups varied (Table 1); seven studies referred to control groups as ‘treatment as usual’ or some form of ‘standard care’ [30, 43, 49, 52, 55–57]. Two studies [45, 58] used waiting list control groups. A clinical guideline posted to GP’s was used as a control in the study by Becker et al. [44]. Pamphlets were used as a control in the study by Brosseau et al. [46] and a copy of the ‘Arthritis Help book’ was given to controls in the study by Hughes et al. [50]. Two studies used self-management programmes in their intervention, but provided it as a stand-alone intervention for controls; [31, 47]. Two studies directly compared two forms of back rehabilitation programmes of varying intensity and content [42, 54]. In the study by Williams et al. [32] the control booklet content differed to the intervention booklet. Krein et al. [53] provided controls with an uploading pedometer and reminder emails to upload data but not access to the web-based intervention, available to the intervention group. In two studies [48, 51] in addition to exercise classes, intervention groups received additional intervention components.

### Outcome measures

Across the 20 studies 13 scales or tools for measuring PA were identified (Table 1) twelve studies reported subjective PA; five objective PA and three reported both. Self-reported measures of PA included estimates of total PA and estimates of frequency, intensity and time in different domains of activity. Only two tools were used in more than one study; the International Physical Activity Questionnaire, [32, 51, 52], and the Freiburg Questionnaire of PA, [44, 54]. Objective measures of PA included steps per day or total PA and/or time in different intensities of PA, measured by accelerometers and/or pedometers.

### Follow-up (post randomisation) (Table 1)

The longest follow up was 18 months [46] six months after a twelve month intervention. Eleven studies reported outcomes at 12 months [42–45, 48, 50, 52–54, 56, 57] however, the latter two studies involved interventions that lasted the 12 months. Four studies reported outcomes at 6 months [30, 31, 51, 58] and one at 3 months [32]. One study had only post-intervention outcomes at four weeks [49] and one study reported outcomes at nine months [47]. Pisters et al. [55] reported outcomes at 65 weeks, the intervention duration was described as 12 weeks however booster sessions were provided to participants up until week 55.

### Risk of bias in included studies (Figs. 2 and 3)

**Fig. 2:**
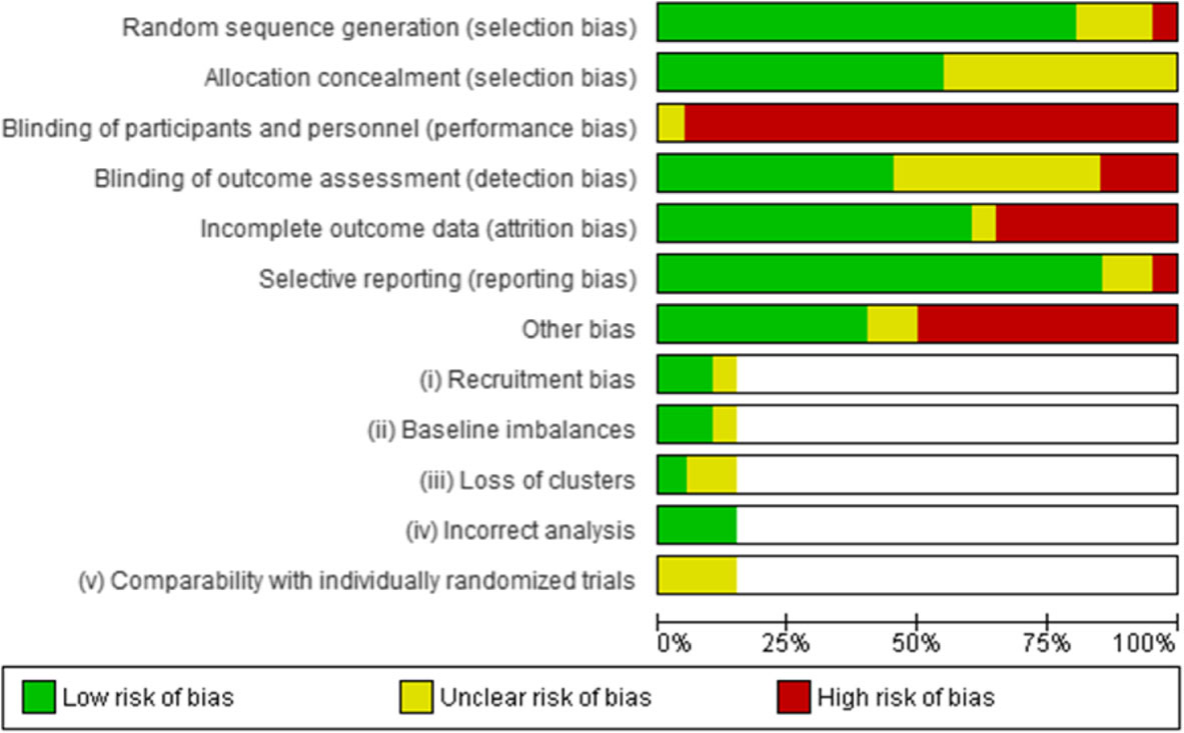
Risk of bias summary of all studies assessed using Cochrane risk of bias tool

**Fig. 3:**
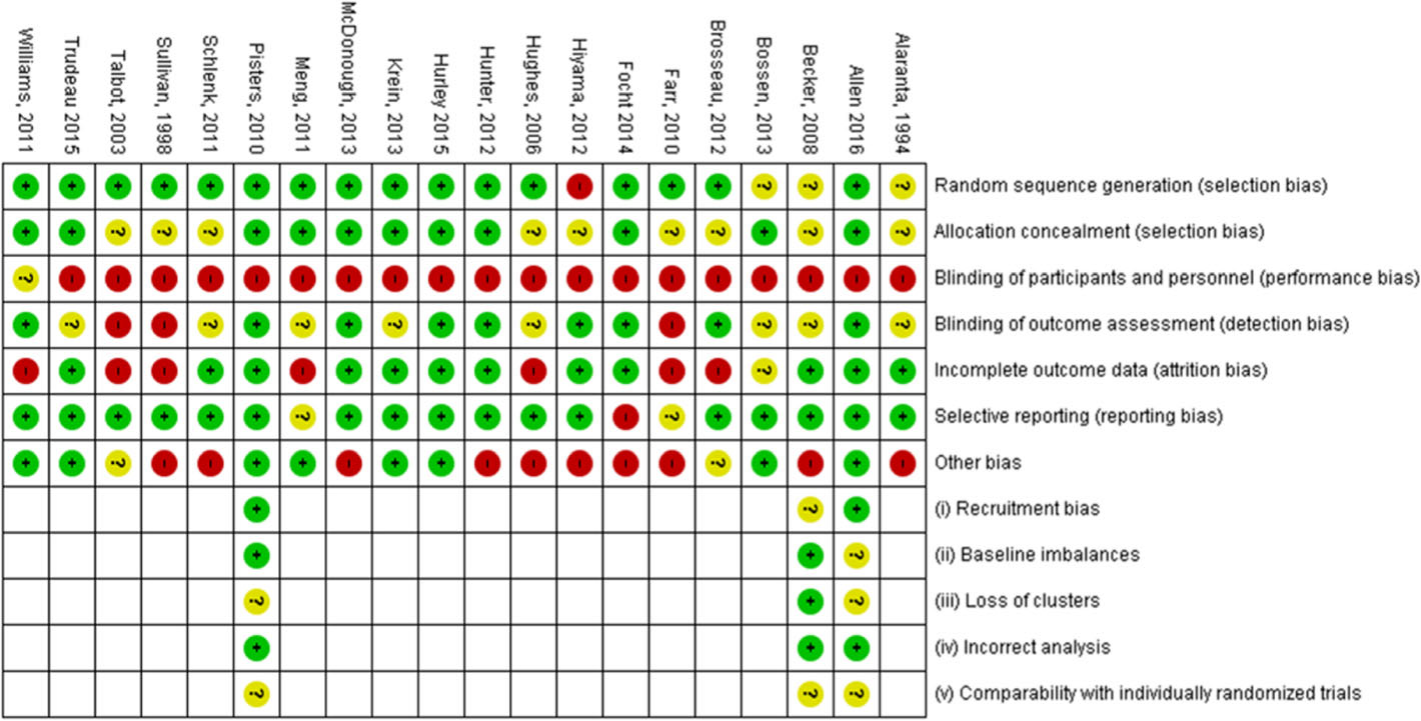
Risk of bias in individual studies

The ROB in the included studies is summarised in Figs. 2 and 3. Blinding, inadequately powered studies and attrition bias were considered the greatest ROB in the included studies. Due to the difficultly in blinding participants and providers in PA interventions, the risk of performance bias was considered high in all but one study which involved posting an intervention or control pamphlet to participants [32], the review authors felt there was insufficient information in the report to support a judgement of high or low ROB for this study. The majority of studies included in the review were not sufficiently powered, only nine reported conducting a power calculation for their primary outcome [32, 43–45, 48, 52–55]. Only two studies [45, 55] conducted power calculations for PA outcomes. Attrition bias was considered high in just over one third of the included studies (35%).

### Risk of bias in cluster randomised controlled trials

Three studies utilised cluster RCTs [43, 44, 55], summarised in (Figs. 2 and 3). Two studies [43, 55] were judged to be of unclear ROB in relation to loss of clusters, this was due to the loss of clusters not being reported or discussed in the analysis or results. ROB on comparability with individually randomised trials was unclear in all three studies, this was largely due to a lack of reporting of comparability or the influence of clustering on intervention effects.

### Effects of interventions: Meta-analysis

Meta-Analysis 1: Effects of Intervention versus control on subjectively measured PA.

Fifteen studies reported continuous measures of subjective self-reported PA [30, 32, 42–46, 50–52, 54–58].

**Short term:** no longer than 12 weeks post randomisation.

**Fig. 4:**
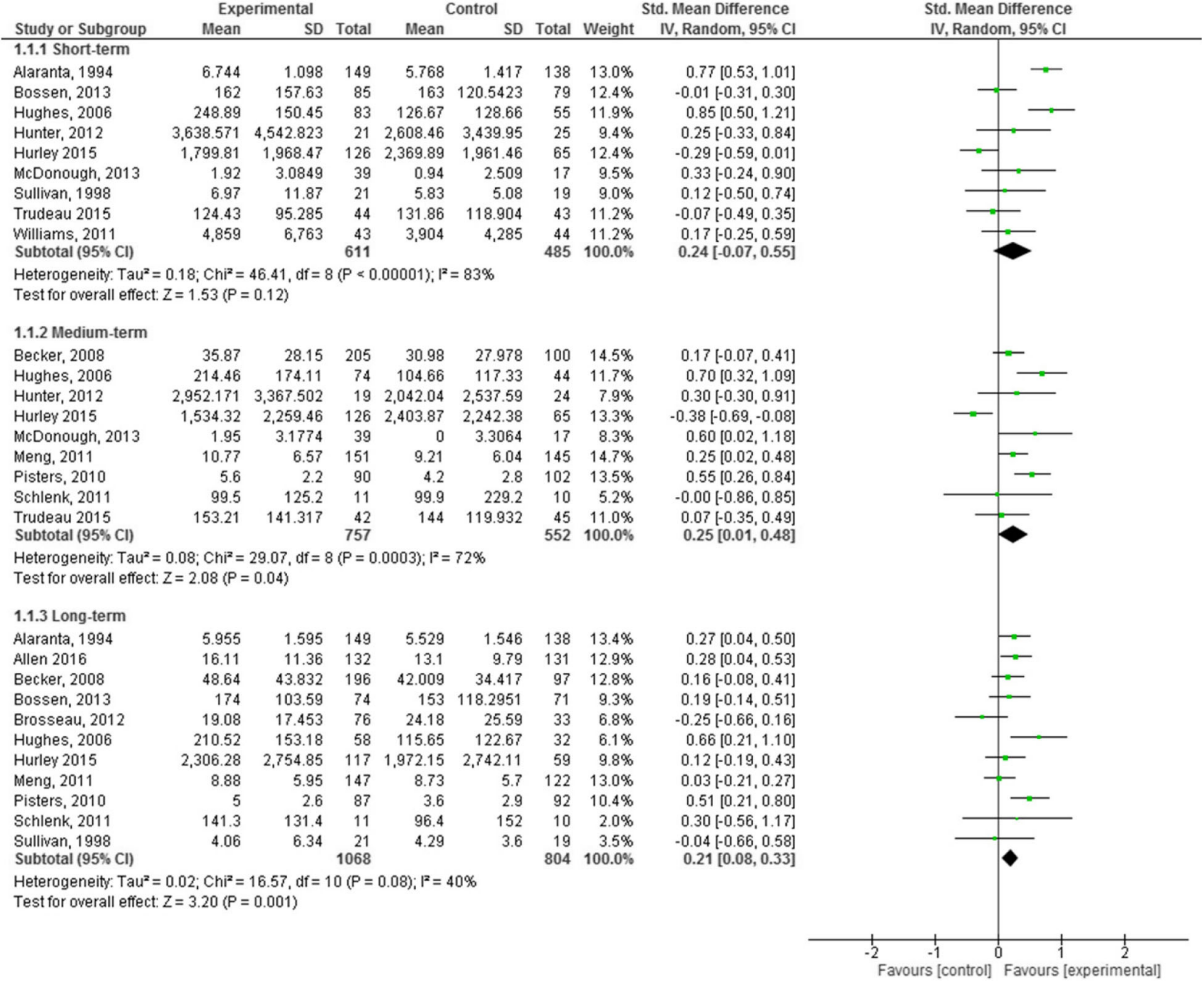
Forest plot of comparison: 1 Effects of intervention versus control on subjectively measured physical activity: short-term, medium-term and long-term

Nine studies (1096 participants) reported short term subjective PA outcomes (Fig. 4) [30, 32, 42, 45, 50–52, 57, 58]. Based on very low quality evidence the pooled effects of the interventions showed no demonstrable effect (SMD 0.24, 95% CI -0.07, 0.55). The quality of the evidence was downgraded from high to very low quality due to substantial statistical heterogeneity (I^2^ = 83%), wide confidence intervals around the effect estimate and ROB (Table 3).

**Table 3:** Summary of quality of evidence using the GRADE approach

Quality assessment							№ of patients		Effect	Quality
№ of studies	Study design	Risk of bias (a)	Inconsistency (b)	Indirectness (c)	Imprecision (d)	Other considerations (e)	Interventions	control	Absolute(95% CI)	
Short-term Subjective Physical Activity										
9	randomised trials	serious	serious	not serious	serious	none	611	485	SMD 0.24 SD higher (−0.07 lower to 0.55 higher)	⨁◯◯◯VERY LOW
Medium-Term Subjective Physical Activity (follow up: range 12 weeks to 6 months)										
9	randomised trials	serious	serious	not serious	not serious	none	757	552	SMD 0.25 SD higher (0.01 higher to 0.48 higher)	⨁⨁◯◯LOW
Long-Term Subjective Physical Activity (follow up: >6 months)										
11	randomised trials	serious	not serious	not serious	not serious	none	1068	804	SMD 0.21 SD higher(0.08 higher to 0.33 higher)	⨁⨁⨁◯MODERATE
Short-Term Objective Physical Activity										
7	randomised trials	serious	serious	not serious	serious	none	255	186	SMD 0.31 SD higher(−0.11 lower to 0.74 higher)	⨁◯◯◯VERY LOW
Medium-Term Objective Physical Activity (follow up: range 12 weeks to 6 months)										
4	randomised trials	not serious	not serious	not serious	very serious	none	135	110	SMD −0.02 SD lower(−0.40 lower to 0.36 higher)	⨁⨁◯◯LOW
Long-Term Objective Physical Activity (follow up: range 6+ months)										
4	randomised trials	serious	not serious	not serious	serious	none	251	184	SMD 0.22 SD higher(−0.02 lower to 0.46 higher)	⨁⨁◯◯LOW

*CI* Confidence interval, *SMD* Standardised mean difference

a. Risk of Bias – Using weighting shown in RevMan analysis a serious downgrade is applied where 25% or more of the results are derived from studies judged to be at high risk of bias (see methods for details), a very serious downgrade is applied where 50% of weighting is derived from studies at high risk of bias

b. Inconsistency – a serious downgrade was applied if there is substantial statistical heterogeneity indicated by an (I^2^) of 50 to 90%. A very serious downgrade is applied if there was substantial heterogeneity and there was inconsistency arising from the populations, interventions or outcomes

c. Indirectness – a serious downgrade is applied if there was indirectness in one of population, intervention, comparator or outcome. A very serious downgrade was applied if there was indirectness in more than one area

d. Imprecision –a serious downgrade is applied when the total population size is less than 400 (provided there is more than one study). Or, if the 95% CI includes 0 (no effect) or the upper and lower confidence interval cross an effect size (SMD) of 0.5 in either direction. A very serious downgrade is applied where there is a small population and imprecision of the effect estimate

e. Where there was sufficient papers (10) a funnel plot was prepared and inspected, a serious downgrade was applied if this suggested a publication bias

**Medium term**: greater than 3 months, not more than 6 months post randomisation.

Nine studies (1309 participants) reported medium term measures (Fig. 4) [30, 44, 50–52, 54–56, 58]. Based on low quality evidence the pooled effects of the studies at the medium term was significant with a small effect size (SMD 0.25, 95% CI 0.01, 0.48). The quality of the evidence was downgraded from high due to the substantial heterogeneity in the observed effects (I^2^ = 72%) and weighting of studies at high ROB included in the analysis (Table 3).

**Long term:** greater than 6 months post randomisation.

Eleven studies (1872 participants) reported long term follow-up measures (Fig. 4) [42–46, 50, 52, 54–57]. Based on moderate quality evidence the pooled effects were small and statistically significant (SMD 0.21, 95% CI 0.08, 0.33) heterogeneity was moderate in the observed effects (I^2^ = 40%). The quality of the evidence was downgraded from high to moderate due to the weighting applied to studies judged as high ROB in the analysis (Table 3).

### Meta-analysis 2: Effects of intervention versus control on objectively measured PA

Eight studies reported objective measures of PA [30, 31, 45, 47–49, 51, 53].

**Short term:** no longer than 12 weeks post randomisation.

**Fig. 5:**
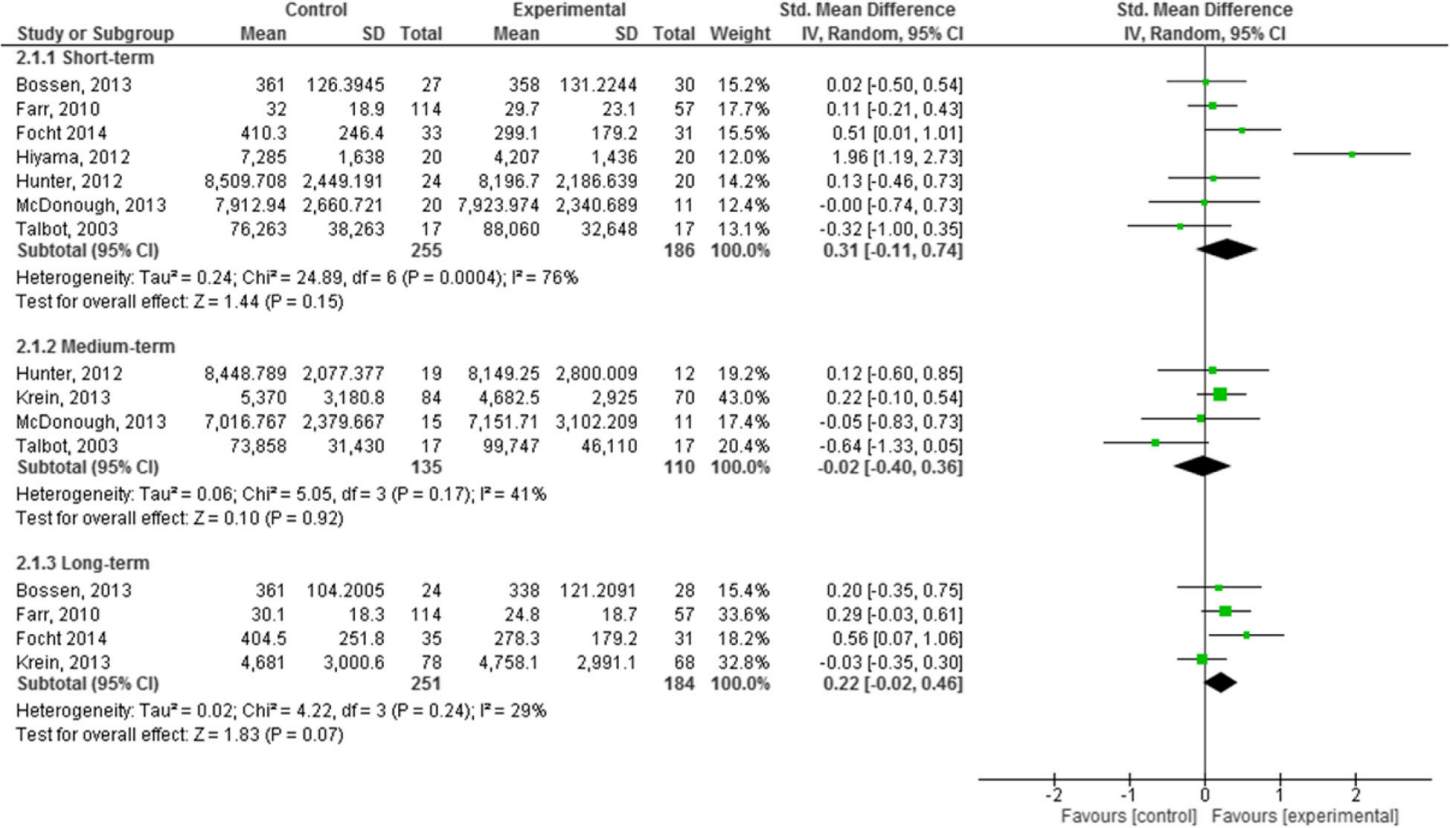
Forest plot of comparison: 2 Effects of intervention versus control on objectively measured physical activity: short-term, medium term and long-term

Seven studies (441 participants) reported short term measures (Fig. 5, Table 3) [30, 31, 45, 47–49, 51]. Based on very low quality evidence, the pooled effect was positive but not significant (SMD 0.31, 95% CI -0.11, 0.74) with substantial heterogeneity (I^2^ = 76%). The quality of the evidence was downgraded from high to very low due to wide confidence intervals in the effect estimates and the weighting applied to studies judged as high ROB in the analysis (Table 3).

**Medium term**: greater than 3 months, not more than 6 months’ post randomisation.

Four studies (245 participants) reported medium term measures (Fig. 5) [30, 31, 51, 53]. Based on low quality evidence, the pooled effect was negative (SMD -0.02, 95% CI -0.40, 0.36) with moderate heterogeneity in the observed effects (I^2^ = 41%). The quality of the evidence was downgraded due to the small number of participants included in the analysis and wide confidence intervals that included no effect.

**Long term:** greater than 6 months post randomisation.

Four studies (435 participants) reported long term follow-up measures (Fig. 5) [45, 47, 48, 53]. Based on low quality evidence, the pooled effect was positive but not significant (SMD 0.22, 95% CI -0.02, 0.46) with low heterogeneity in the observed effects (I^2^ = 29%). The quality of the evidence was downgraded from high to low due to imprecision of the effect estimates as evidenced by the confidence intervals included no effect and the weighting applied in the analysis to studies at high ROB.

### Sensitivity analysis

We examined the pooled effects for the two types of outcomes (subjective and objective) at each time point by an assessment of the ROB. When limited to studies with a lower ROB, effect sizes were not significant at any timepoint.

**Subgroup Analyses:** To increase statistical power for the planned subgroup analysis we used subjective measures of PA (*n* = 16 studies).

**Subgroup analysis 1:** Clinical conditions osteoarthritis and low back pain:

Effects were demonstrated for the osteoarthritis subgroup only, effects sizes were moderate in the medium-term (SMD 0.41, 95% CI 0.10, 0.72) and small in the longer term (SMD 0.29, 95%CI 0.08, 0.49).

**Subgroup analysis 2:** Intervention Intensity:

Only interventions that were of higher intensity, relative to the median calculated contact hours of the interventions (8.3 h) reached important effect sizes (seven studies). Higher intensity interventions resulted in moderate effect sizes for short term (SMD 0.66 95% CI 0.41, 0.91) and medium term (SMD 0.47 95% CI 0.20, 0.74) outcomes, and small effect sizes for longer term outcomes (SMD 0.25 95% CI 0.02, 0.48).

### Influence of BCTS and recruitment route

**Fig. 6:**
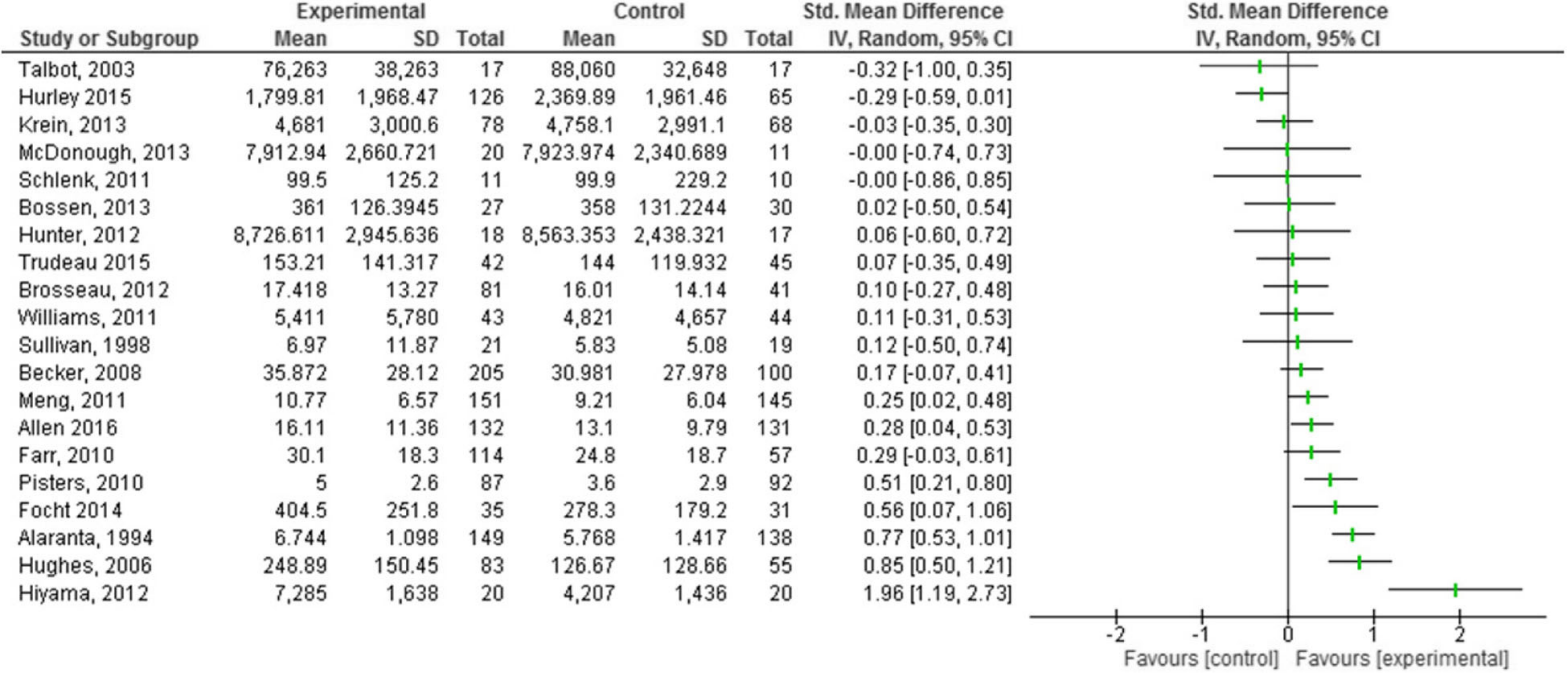
Forest plot: Studies grouped by effect size (aggregated subjective and objective measures) post intervention

It was not possible to conduct the quantitative subgroup analysis of BCTs and recruitment routes as the data generated from the review was not sufficient to permit valid comparisons. Descriptive statistics were used to describe possible associations between these factors and other intervention characteristics. To facilitate this exploration, all studies were grouped by effect size, post intervention (Fig. 6).

### Behaviour change techniques

Seven studies demonstrated statistically significant small to large effect sizes on post intervention PA (Table 2). Across these studies, 60 BCTs were coded with a mean of 8.57 per study, range (1–16). In total 28 unique BCTs were identified, the most commonly coded were ‘goal setting behaviour’, and ‘instruction on how to perform the behaviour’ featuring in 71.4% of studies. ‘Self-monitoring behaviour’, ‘social support (unspecified)’, and ‘framing/reframing’ were also coded frequently and were present in over half of the included studies (57%).

Thirteen studies demonstrated no effect, or negligible effects (<0.2) post intervention (Table 2). Across these studies 100 BCTs were coded with a mean of 7.7 per study, range (0–15) with 31 unique BCTs present. The most commonly coded BCTs were; ‘goal setting behaviour’, ‘information on health consequences’ ‘instruction on how to perform the behaviour’ and ‘behavioural practice/rehearsal’ which featured in 61.5% of the studies.

### Recruitment route and other intervention characteristics: (Tables 1and 2)

No notable differences were observed with regards to the influence of recruitment route, type of PA, mode of delivery and post-intervention effect sizes.

In seven studies demonstrating positive effects, five (71.4%) were delivered by healthcare professionals (2 multidisciplinary and 3 by physiotherapists). In comparison, studies with no effect (<0.2) were less frequently delivered by healthcare professionals (53.8%).

## Secondary outcomes

### Adverse incidents

Only six studies made explicit statements regarding adverse incidents; two studies, although not explicitly stated, documented adverse incidents. Allen et al. [43] reported four adverse incidents unrelated to the intervention; one study [51] reported no adverse incidents related to the exercise components. Relatively minor musculoskeletal complaints were reported in three studies [30, 52, 53]. Allergic reactions to pedometer clips [30] and minor cardiovascular events [53] were also reported. One author [52] noted that half of the participants in a walking group who developed increases in musculoskeletal complaints withdrew from the study. A fall resulting in a hip fracture sustained during a session was reported in one study [57] and three withdrawals due to increasing back pain were reported [42].

## Discussion

### Summary of findings

This is the first systematic review and meta-analysis examining the effectiveness of interventions in improving subjective and/or objective levels of PA in adults with PMP and possible associations between BCTs and other intervention characteristics on effect sizes.

In builds on the findings of two similar reviews; Williamson et al. [60] who assessed the effectiveness of behavioural PA interventions in participants with lower-limb osteoarthritis, and Oliveira et al. [33] who assessed the effectiveness of interventions in increasing objectively measured PA in chronic musculoskeletal pain. In contrast to the latter study this review makes a clear distinction between therapeutic exercise programmes and interventions specifically aimed at increasing PA levels or ‘habitual PA behaviours’.

With respect to subjective PA, interventions were ineffective in the short term (up to 12 weeks, very low quality evidence); or had a small effect medium term (3–6 months: SMD 0.25, 95%CI 0.01 to 0.48, low quality evidence) and long term (SMD 0.21 95% CI 0.08 to 0.33, moderate evidence). Given the quality of the evidence further research is likely or very likely to have an important impact on our confidence in the estimate of effect and is likely to change the estimate. Analysis of the evidence for objective outcomes showed that interventions were not effective at any time point. These observations were based on very low to low quality evidence therefore the estimate of effect is very uncertain and further research is very likely to change the estimate.

Subgroup analyses indicated that interventions were more effective in improving PA levels in adults with osteoarthritis compared to those with persistent low back pain. Intervention effects were also consistently higher in interventions with a greater number of contact hours (> 8.3 h). These subgroup analyses should be interpreted with caution; as differences may not relate to their classifications. However, subgrouping participants by condition was clinically plausible and intervention intensity has previously been associated with effectiveness.

### Comparison of subjective outcomes with published literature

Two reviews examining long term outcomes of PA interventions: a Cochrane review of face-to-face interventions to promote PA [61] and a systematic review of PA interventions for adults aged 55–70 years [62]: both reported significant, but very small effects (SMD 0.19) at 12 months. Similarly, this review found small effects for outcomes measured beyond six months (SMD 0.21 95% CI 0.08, 0.33). These findings may indicate that individuals with pain respond to PA interventions in a similar manner to non-pain populations.

In a subgroup analysis Williamson et al. [60] found intervention effects were greatest between 6 and 12 months (SMD 0.53, 95% CI 0.41 to 0.65) and that the effectiveness of interventions declined over time, reporting no significant benefit compared to controls in outcomes beyond 12 months. Similarly, in our osteoarthritis sub-group we found a moderate effect size for medium term outcomes (>3 months ≤6 months) (SMD 0.41, 95% CI 0.10, 0.72) that diminished over time (>6 months) (SMD 0.29, 95% CI 0.08, 0.49). These findings may suggest that individuals with osteoarthritis make changes to their PA levels gradually. However, without ongoing support or increased efforts directed towards maintenance of PA, individuals with osteoarthritis may struggle to sustain increased levels of PA.

### Comparison of objective outcomes with published literature

In line with our own findings of no detectable effect on objectively measured PA, Oliveira et al. [33] also found no effect on short, intermediate or long term objective outcomes. Williamson et al. [60] were unable to conduct a meta-analysis using objective measures due to a lack of studies reporting objective measures. In contrast to our findings, the review of interventions aimed at increasing PA in adults aged 55 to 70 years, found larger effects for objective measures (steps per day) (SMD 1.08; 95%CI 0.16, 1.99) at 12 months [62]. A possible explanation for this difference could be that the participants included in this review by Hobbs et al. [62] were essentially ‘healthy populations’ in contrast, our review and that of Williamson et al. [60] and Oliveira et al. [33] all involved participants with PMP.

### Intervention characteristics

We found interventions with a higher number of contact hours resulted in greater effect sizes. Similarly in a post hoc meta-regression, Williamson et al. [60] also found, that a higher number of contact hours had a significant influence on intervention effectiveness. In contrast Hobbs et al. [62] found less intensive interventions were more effective than higher intensity interventions. A plausible explanation for these contrasting findings, is that those with PMP may need additional interventional support, in order to successfully change their PA behaviours in comparison to healthy populations.

In this review the influence of BCTs on PA outcomes is unclear but the findings are consistent with those of previous reviews. Bishop et al. [63] published a review and meta-analysis exploring the effects of contextual and BCT content of control and target interventions in 42 trials included in a Cochrane review of interventions to improve adherence to exercise for chronic musculoskeletal pain [64]. In keeping with the findings from our review, among the most frequently coded BCT’s were ‘instruction on how to perform the behaviour’ and ‘behavioural practice and rehearsal’. A finding also reported by Keogh et al. [65] who reviewed BCTs utilised in chronic low back pain self-management programmes. We found ‘self-monitoring of the behaviour’ was amongst the most frequently coded techniques in interventions with greater effect sizes, a finding not replicated in the either the Bishop et al. [63] or the Keogh et al. [65] reviews, but consistent to findings of PA reviews in healthy populations [24], older adults [66], and in obese adults [28]. As our review was more narrowly focused on habitual PA as opposed to adherence to exercise or self-management, this finding (although tentative) lends some support to the evidence that this technique may be particularly useful in PA interventions.

Interventions included in this review were generally multifaceted often involving several modes of delivery with varying degrees of complexity. It was difficult to draw firm conclusions regarding which characteristics of interventions are associated with more effective interventions.

Few studies provided explicit statements regarding adverse incidents; where they were reported they were largely limited to minor musculoskeletal complaints. Although risk of adverse incidents in PA interventions is generally regarded as low; it is plausible that exacerbating pain may have a deleterious effect on participation, particularly in those with PMP.

### Completeness and quality of the evidence

The quality of the evidence within this review ranged from moderate to very low across the different time-points and outcomes. Effect sizes at best are small and limited to subjective measures. Key limiting factors leading to downgrading the quality of the evidence were, ROB, statistical heterogeneity in the observed effects and imprecision as evidenced by wide confidence intervals. With respect to ROB many studies were designed to identify changes in pain and function/disability as their primary outcomes and were thus underpowered to detect changes in physical activity levels; as such the results of this review should be interpreted with caution. Furthermore, a number of studies failed to provide adequate detail regarding blinding of outcome assessors and allocation concealment. In cluster randomised controlled trials it was often unclear if authors had considered the effect of trial design and the influence clustering may have had on results and whether this was considered when comparing effects with other trials.

Whilst the use of validated measures of PA, was in itself a strength, a more standardised approach to reporting PA data would have permitted a more robust statistical analysis, strengthening the evidence. Self-report measures are known to be prone to recall bias: it has been suggested that as both the intervention and control groups complete the measure any misclassification should be non-differential [67]. However, it could be argued, that using self-report measures in interventions where participants and providers are also unlikely to be blinded the potential of recall bias is increased. Only three studies included subjective and objective measures; this approach might be considered ideal given the relative strengths and limitations of each.

Descriptions of intervention content varied greatly impacting on the number of BCTs that could be reliably reported as occurring within an intervention. In this review we only coded BCTs clearly delivered to the participants and directed towards the target behaviour. As reported by others, [24, 63] this approach, although more rigorous, may result in less BCTs being coded than were actually delivered.

The variation noted across the control conditions could have influenced effect-estimates with smaller between group effects associated with comparisons against more active control treatments [68]. However, we did not detect this when reviewing individual effect size comparisons.

Six studies initially assessed as suitable for inclusion did not report means, standard deviations or sample sizes and requests to obtain this data from study authors were unsuccessful; this data could have added to the quality of the evidence in this review.

Study participants were largely recruited from primary or secondary care (General Practitioners, physiotherapy clinics): it is very possible that the effects seen in those recruited via these settings, differ to those accessing for example, specialist pain services.

### Potential biases in the review process

Studies were primarily excluded from the review because a suitable measure of PA was not reported. This may reflect a selective reporting bias; however, it is suggested this is more likely to reflect the changing emphasis of healthcare interventions, particularly the drive towards self-management and a public health approach to managing long term conditions. Although databases were searched from inception only two studies included in the review were published prior to 2003 [42, 57].

## Conclusions

### Implications for practice

Based on the findings of this review it is not possible to conclude which characteristics of interventions are more effective. However, based on observational analysis and in line with findings of previous reviews, integration of behavioural techniques such as; ‘self-monitoring of the behaviour’, ‘instruction on how to perform the behaviour’ and ‘goal setting (behaviour)’ may be indicated. Higher intensity interventions - in terms of the estimated contact time with the intervention, may be more effective than less intensive interventions.

The emphasis of PA and exercise interventions in PMP has largely been directed at reducing pain and disability. However, these interventions may have little impact on the overall level of PA an individual engages. Targeted behaviour change interventions are likely to be required to address the risk of morbidity and mortality in this population.

### Implications for research

Persistent pain, like many other non-communicable diseases is influenced by several determinants of health such as; socioeconomic status, education, employment and mental health [69]. There is a need for future studies to adopt methods to encourage and secure participation from individuals representing the broad spectrum of persistent pain patients. In particular, those accessing specialist pain services were under represented in this review. Individuals accessing specialist pain services are often deemed to be on the more severe end of the pain spectrum and typically report much higher levels of disability and poorer health related quality of life scores [2]. We agree with previous suggestions [70] that health inequalities may actually be increased because of differences in responses to recruitment. A clear finding from this review is the need to standardise the measurement of PA in PMP populations.

To improve the quality of evidence, future studies should be sufficiently powered, collect longer term follow up data and report on cost-effectiveness. Study authors should report methods for blinding outcome assessors clearly. Providing access to supplementary data such may improve the quality of coding and reporting of intervention content. Future reviews should consider incorporating meta-regression or moderator analysis to explore if specific components or characteristics of interventions are associated with more effective interventions.

### Differences between published protocol and review

The review authors reappraised the decision to include unpublished studies and included only those that had been published.

Study authors were amended: SH was added to review team and coded intervention content. LA was added to the review team and provided expert input on aspects relating to coding of BCTs.

The review team agreed to limit the extraction of secondary outcomes to adverse incidents relating to the intervention. There were two main reasons; firstly, to maintain the focus and specificity of the review. Secondly a number of systematic reviews have recently been published describing many of the secondary measures; pain, disability and function, it was felt that extracting these outcomes would be of little additional value to readers of the review.

ROB: The validity of the PA outcome measure is not added as an additional domain within the ROB. This data was included in the data extraction forms and is discussed in relation to outcome measures. An additional domain of sample size calculation for the primary outcome (not specifically for PA) was added to the ROB table and a priori agreements were made during piloting of the ROB table with regards to agreed cut-offs for attrition bias.

The GRADE approach was adopted post-protocol to rate the quality of evidence generated within the review process.

## Additional files

Additional file 1: Search Strategy for Medline. (DOCX 13 kb)

## Abbreviations

BCT: Behaviour change technique
GRADE: Grading of recommendations, assessment, development and evaluations
PA: Physical Activity
PABAK: Prevalence-adjusted bias-adjusted Kappa
PMP: Persistent musculoskeletal pain
RCT: Randomised controlled trials
ROB: Risk of bias
SMD: Standardised mean difference
